# A modular PLC simulation method for virtual replication of a cost-effective industrial automation laboratory^☆^

**DOI:** 10.1016/j.mex.2026.103864

**Published:** 2026-03-14

**Authors:** Musa Al-Yaman, Dana Alswaiti, Ahmad Birawi, Adham Alsharkawi, Majid Al-Taee

**Affiliations:** a Mechatronics Engineering Department, The University of Jordan, Amman, Jordan; b Department of Electrical Engineering and Electronics, University of Liverpool, Liverpool, UK

**Keywords:** Programmable Logic Controller (PLC), PLC simulation, Modular industrial laboratory, Virtual replication, Educational simulation method

## Abstract

Access to hands-on PLC training is often limited by the cost and complexity of physical automation laboratories, while existing simulation tools typically lack alignment with real hardware configurations, reducing their effectiveness for education. To address this, we present a modular PLC simulation method that enables accurate virtual replication of a cost-effective, scalable industrial automation laboratory used for traffic light control, elevator operation, and automated filling systems. Built in Unity, the method integrates a custom ladder logic execution engine with interactive 3D models that mirror the exact input/output structure and operational behavior of the physical laboratory. Users can program, test, and debug logic in a realistic environment and receive immediate visual feedback—without requiring hardware. The method was validated by comparing its outputs against the physical system across 4 representative automation tasks; in every case, the virtual and physical setups produced I/O sequences matching within ±10ms and control outcomes, confirming functional equivalence.

• Introduces a modular simulation framework that faithfully replicates the application scope of a physical low-cost PLC training laboratory.
• Combines a custom ladder logic interpreter with real-time 3D visualization in Unity to enable program testing and debugging.
• Validates functional equivalence through direct behavioral comparison with physical hardware across 4 standard automation tasks.

## Graphical abstract

**Figure:**
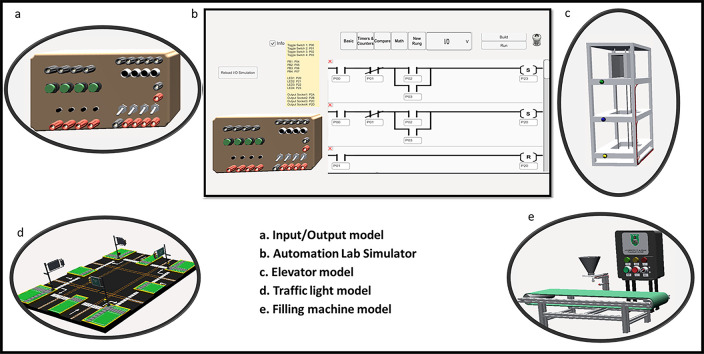

**Specifications table**

**Table:** 

**Subject area**	Engineering
**More specific subject area**	*Mechatronics Engineering*
**Name of your method**	*A PLC Programming Framework for a Digital Industrial Automation laboratory*
**Name and reference of original method**	*A Cost-Effective Modular Laboratory Solution for Industrial Automation and Applied Engineering Education*
**Resource availability**	*Software:* *Unity Engine* *C#*

## Background

Hands-on training in programmable logic controller (PLC) programming is essential for mechatronics engineering education, providing students with practical skills in automation and control. However, physical PLC laboratories are often expensive, require significant maintenance, and may be inaccessible to many institutions [1]. To overcome these challenges, educators increasingly rely on simulation-based alternatives, which offer safe, cost-effective, and scalable opportunities for students to practice programming and validate control logic before interacting with real hardware.

Early efforts, such as the experimental mechatronics setup developed by S. Çeven and A. Albayrak [1], demonstrated how dedicated hardware platforms can significantly enhance learning. At the same time, they highlighted challenges related to cost, scalability and maintenance. To improve accessibility, J.L. Vázquez González et al. [2] proposed simulated laboratories that allow students to safely experience extreme or rare industrial conditions. Similarly, D. Popescu et al. [3] pointed out that direct experimentation on physical systems is often impractical, suggesting simulation as a more economical and flexible solution.

Building on these ideas, W.-J. Shyr [4] developed a virtual PLC laboratory using the Multiprog environment, featuring elevator and racing-light simulations. Although intuitive, the system relied on proprietary software and supported only predefined scenarios. In parallel, J.J. Brito, P. Toledo, and S. Alayón [5] created a “Storage and Distribution Station” by combining mechanical modeling in Autodesk Inventor with control simulation in MATLAB-Simulink. While technically rich, this approach required specialized tools and didn’t support standard ladder logic programming in a native form. Other researchers focused on remote access: P. Chand et al. [6] that allowed students to program real PLCs remotely, though it still depended on physical hardware and stable network infrastructure. Likewise, M. Abdulwahed and Z.K. Nagy [7] introduced TriLab, a blended framework combining virtual, remote, and hands-on modes via LabVIEW and Joomla. Despite its pedagogical ambition, its complexity limited large-scale deployment.

A common limitation across most existing simulators is the lack of native replication of the exact input/output (I/O) addressing architecture used in educational PLC kits [8,9]. Although commercial and open-source platforms allow variable remapping, this typically requires manual configuration and network protocol setup for each experiment. Such additional infrastructure complicates implementation and prevents direct transfer of ladder logic code from simulation to hardware without modification. As a result, the continuity of learning is disrupted, and student confidence in virtual training may be reduced.

To address this gap, we propose a modular PLC simulation method that functions as a hardware-aligned virtual counterpart to our previously published low-cost physical automation laboratory [10]. The original hardware platform supports foundational applications such as basic I/O control, traffic light sequencing, elevator operation, and automated filling systems. In the present method, the physical kit’s I/O mapping is embedded directly into a Unity-based 3D environment, with plant models developed in Blender. This architecture enables zero-configuration code portability: students can write ladder logic, validate it against realistic plant dynamics in simulation, and deploy the same program to the physical hardware without address translation.

Unlike generic simulators designed for broad industrial flexibility, the proposed method is intentionally tailored for integrated educational use. By preserving exact I/O correspondence between virtual and physical environments, it ensures that the mental model formed during simulation transfers seamlessly to real hardware. The system is modular, extensible through configurable JSON mappings, and validated for functional equivalence, offering a practical and reusable solution for PLC education worldwide.

## Method details

### Methodological positioning and comparison

To realize the hardware alignment described above, the proposed method replicates the modular, cost-effective PLC laboratory introduced in [10], supporting four core experimental modules: (i) basic I/O training, (ii) elevator control, (iii) traffic light sequencing, and (iv) automated filling system operation. Each module preserves the same input/output addressing scheme as the physical kit, ensuring structural and functional equivalence between the virtual and real systems.

Although commercial platforms such as CODESYS [11] and Factory I/O [12] provide powerful and industry-grade simulation capabilities, they are primarily designed for industrial flexibility rather than strict educational hardware alignment. In typical implementations, integrating these tools with educational PLC hardware requires manual variable mapping, communication interface setup, and network protocol configuration (e.g., OPC UA). For introductory-level students, this additional configuration layer can introduce unnecessary cognitive load, shifting attention away from fundamental control logic concepts toward infrastructure management.

In contrast, the proposed method is intentionally designed around zero-configuration code portability. Ladder logic developed and tested within the simulation environment is executed on the physical PLC hardware without any address translation, remapping, or modification. By eliminating intermediary configuration steps, the approach preserves continuity in the learning process and reinforces a consistent mental model of PLC I/O behavior. Table 1 summarizes the key methodological distinctions between the proposed hardware-aligned approach and integration-based alternatives, focusing on dimensions critical to scalable and accessible educational deployment.

**Table 1:** Comparative analysis of simulation approaches for educational PLC laboratories.

Feature	Proposed Method	Commercial Integration (CODESYS + Factory I/O)	Generic Open-Source Integration (e.g., OpenPLC)
Primary Focus	Introductory education & hardware alignment	Industrial training & flexibility	Open-source flexibility
I/O Mapping	Embedded JSON configuration (matches physical kit addresses)	Manual variable tagging & OPC mapping	Manual address table configuration
Code Portability	Byte-for-byte transfer (no modification)	Requires project reconfiguration per stage	Requires address synchronization
Communication	Internal function calls (no network)	Network protocol (OPC UA/Modbus TCP)	Network protocol (Modbus TCP)
Setup Complexity	Single executable (zero configuration)	Multiple runtimes + driver configuration	Multiple runtimes + dependency management
Cost	Free (academic development)	Commercial licenses (∼$1,500+)	Free (open-source)
Pedagogical Advantage	Reduced cognitive load (focus on logic)	Full industrial workflow exposure	Technical integration skills

### System architecture

The proposed system architecture is designed to guarantee functional equivalence between the virtual simulation and the physical laboratory. In practice, this means that any ladder logic program developed and validated in the virtual environment produces functionally equivalent input/output (I/O) behavior when deployed on the actual hardware, without requiring modification, remapping, or reconfiguration.

**Fig. 1:**
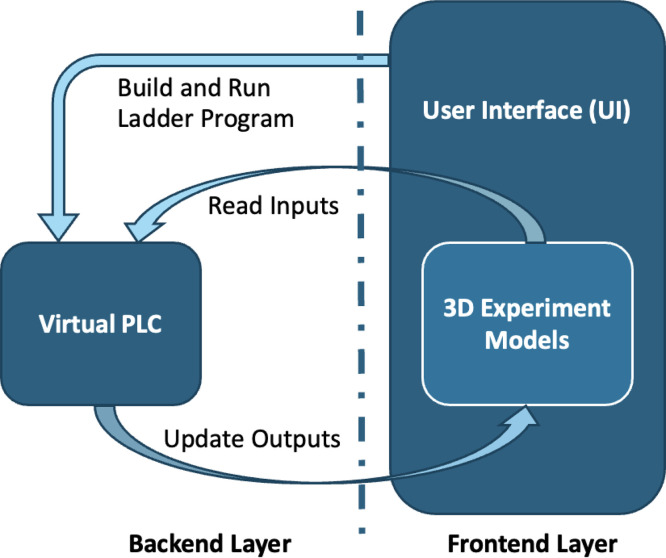
System architecture showing the virtual PLC engine (backend) and 3D experimental modules (frontend), with bidirectional I/O state exchange.

As shown in Fig. 1, the system is structured into two closely integrated yet functionally decoupled layers; frontend and backend. This separation ensures that the execution of ladder logic remains independent of the visual representation, preserving the integrity of I/O operations while enabling realistic, interactive simulation. The roles and components of each layer are described in detail below.

• Backend layer: A virtual PLC execution engine responsible for interpreting ladder logic, managing the scan cycle, and updating I/O states according to the embedded hardware-aligned addressing scheme.
• Frontend layer: An interactive 3D models and a user interface that visualize plant behavior and enable user interaction with virtual sensors and actuators.

### Backend layer: Virtual PLC execution engine

The backend layer implements a virtual PLC that replicates the cyclic scan behavior of industrial controllers and ensures full compatibility with the I/O architecture of the physical laboratory described in [10]. The engine operates in three phases during each scan cycle:

(1) Input scanning: reads the current states of all digital inputs from the active 3D experiment model;
(2) Logic execution: evaluates user-defined ladder logic using a custom interpreter;
(3) Output updating: applies the resulting output states to drive actuators and indicators in the simulation.

**Fig. 2:**
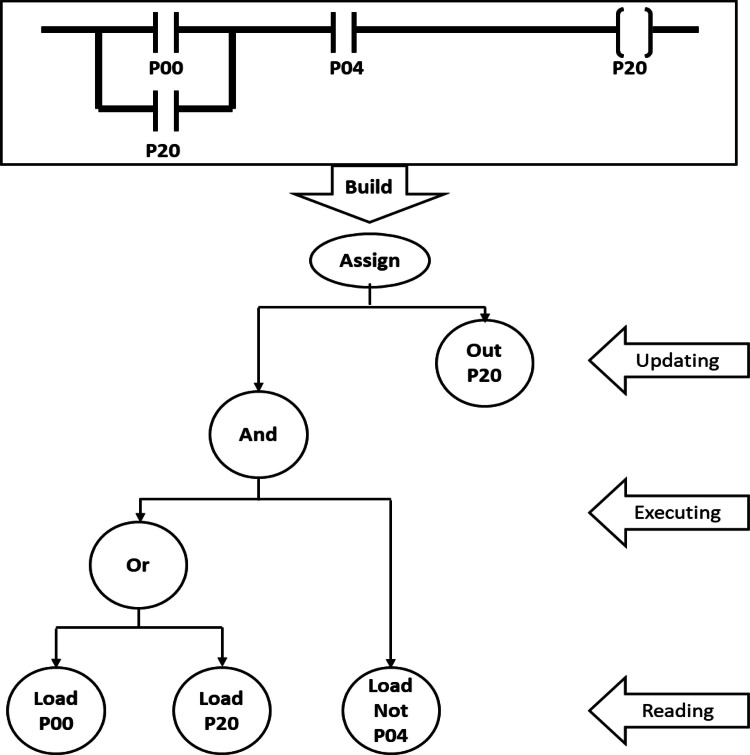
Internal binary tree representation of a ladder logic rung, used for efficient logical evaluation during the scan cycle.

The ladder logic interpreter processes programs as plain-text files containing standard relay instructions (e.g., XIC, XIO, OTE, TON). Each instruction is parsed according to a tag-based schema that mirrors the programming conventions used with the physical PLC laboratory. For example, parallel branches (OR logic) and series contacts (AND logic) are reconstructed into an internal binary tree representation as illustrated on Fig. 2, enabling accurate evaluation of complex rungs during each scan.

Importantly, the virtual PLC maintains a memory map that exactly replicates the input (I0.x) and output (Q0.x) addressing scheme of the physical laboratory platform [10]. This one-to-one correspondence guarantees that any ladder logic program validated in simulation can be transferred directly to the hardware without address translation, remapping, or structural modification.

Furthermore, particular attention is given to usability and interactivity to enhance the learning experience. After hitting the run button, the run script accesses the program list and reads the current virtual PLC memory states. Then, the script accesses each rung individually going through the instructions and evaluating the outputs. After that, the output is tested, if there is a false, the associated rung output turns false.

**Fig. 3:**
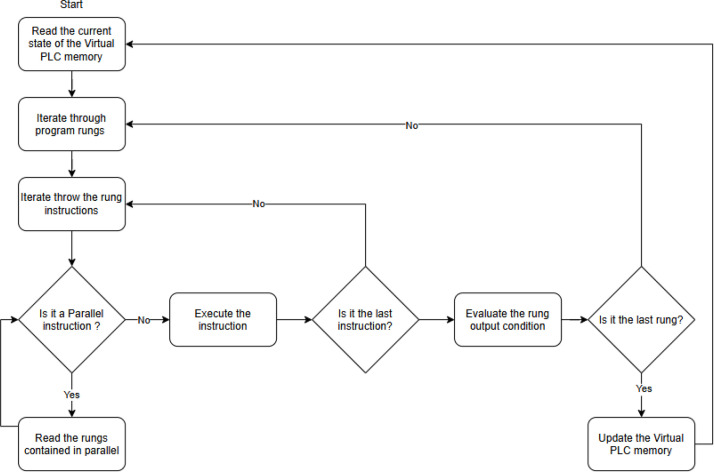
Overview of script execution flow.

Fig. 3 provides a detailed representation of the run-cycle workflow and instruction evaluation process. During execution, bidirectional interaction occurs between the student and the 3D experimental models: user actions (e.g., activating virtual sensors or switches) update the PLC input states, and computed outputs dynamically drive the behavior of actuators and plant components within the simulation environment. This closed-loop interaction reinforces the connection between ladder logic design and observable system response.

### Frontend layer: 3D simulation environment

The frontend provides a real-time, interactive 3D representation of the four experimental modules (basic I/O training, elevator control, traffic light sequencing, and automated filling system) exactly as configured in the physical laboratory [10]. All models were developed in Blender to match the geometry, labeling, and component layout of the hardware rigs, then imported into Unity [13] for simulation.

Each virtual sensor (e.g., push buttons, limit switches, photoelectric sensors) and actuator (e.g., indicator lights, motors, solenoid valves) is programmatically linked to its corresponding I/O address in the virtual PLC’s memory map (e.g., pressing the “floor 2 call button” sets input I1.3 to HIGH; activating output Q0.1 turns on the green traffic light). This one-to-one I/O binding ensures that the simulation responds to logic execution identically to the physical setup.

The user interactions are limited to discrete, state-changing actions (e.g., pressing a button, toggling a switch), which directly modify input states, mirroring how real-world sensors trigger PLC inputs. Actuator responses (e.g., elevator movement, traffic light transitions, fluid filling level) are driven solely by output states from the virtual PLC, updated in real time during each scan cycle. No scripted behaviors or autonomous animations are used; all dynamics emerge from the executed ladder logic. The environment supports seamless switching between experiments without restarting the simulator, preserving modularity consistent with the physical laboratory’s design. Visual feedback is purely functional (focused on clear indication of component states) avoiding decorative elements that could distract from I/O behavior. The frontend interface serves as the primary point of interaction for students, enabling them to engage with the system through several key functionalities:

• Select from multiple 3D experiment modules, each representing a distinct industrial control scenario.
• Construct ladder diagrams using a user-friendly drag-and-drop system, which includes a wide range of programmable logic instructions—from basic input/output controls to advanced timers and arithmetic functions.
• Compile and execute control logic using an integrated virtual PLC, with real-time communication between the ladder logic and the simulation environment.
• Visualize the simulation in real time, observing how changes in logic affect the states of sensors, actuators, and indicators in the 3D experiment setup.

**Fig. 4:**
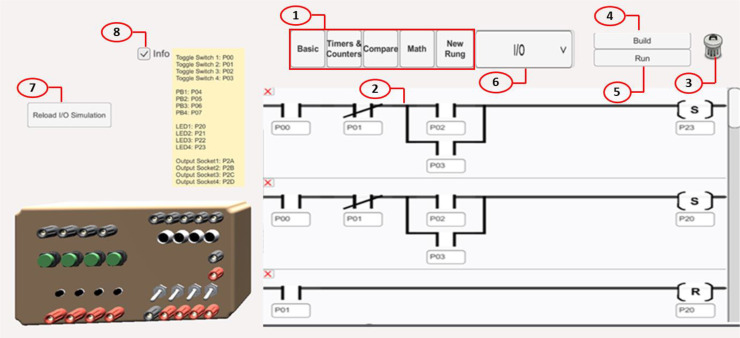
The User Interface of the program.

A user interface is the space where interactions between humans and machines occur. It is implemented using C# language and integrated with the Unity platform [13] that used to build the 3D experiments models. The UI, is the place where the student can select the 3D experiment; write a ladder diagram code that will provide the process’s logic of execution, this will be easily done using the set of instructions; build the code and run the code as seen in Fig. 4. The UI consists of mainly eight components as follows:

1. Programming Menu: it contains drop-down menus for the instructions and a new rung button. It has all instructions from basic input/output commands to timers and mathematical operations. It is built from a panel as a parent and buttons as children. One of those drop-down menus is the basic menu that contains instructions to be used in almost every LD program. It includes instructions that read and change the PLC bit memory in addition to move instruction which is used to change the value of words in PLC. These components can be dragged and dropped on the rungs inside the programming area.
2. Programming Area: it is the window containing the user selected instructions and rungs.
3. Delete icon: it removes the instructions and clear the programming area.
4. Build button: it is used to transfer the code to the virtual plc and do the compilation process.
5. Run button: it is used to start the simulation on virtual plc with data exchanged with the selected 3D experiment model.
6. Select Experiment: the experiment can be selected from the drop-down menu and then shown in the area to the left of the programming area.
7. Reload Simulation button: it is used to restore all the experiment's elements to their original state and position and reset all the bits to their initial values.
8. Info check box: it is used to show the corresponding bit/address for each component.

### Implementation requirements

To support reproducibility and deployment across different institutional contexts, the simulation environment requires the technical specifications summarized in Table 2. These include the Unity version, tested operating systems, hardware requirements, and runtime dependencies necessary for smooth operation.

**Table 2:** Representative I/O transition log for Traffic Light task (North-South Green Light).

Specification	Details
Unity Version	2021.3.15f1
Tested Operating Systems	Windows 11 (64-bit)
Minimum CPU	Intel Core i5
Recommended CPU	Intel Core i7
Minimum GPU	Integrated graphics
Recommended GPU	Dedicated GPU with 2GB VRAM
RAM	8 GB minimum, 16 GB recommended
Storage	2 GB available space
Runtime Dependencies	.NET Standard 2.1, Unity Input System package
Build Target	Standalone Windows

### Extensibility and modularity

The proposed two-layer architecture facilitates the addition of new experimental modules without modifying the backend Virtual PLC engine. To integrate a new experiment, only the frontend 3D model needs to be updated along with its corresponding I/O mapping configuration, which remains fully configurable via a JSON file, e.g., {"virtual": "btn_start", "physical": "%IX0.1"}. Adding a new discrete I/O experiment typically requires ∼4-6 hours for an experienced developer. The current implementation supports discrete (Boolean) I/O only; extending to analog signals or high-speed pulse modules would require modifications to the backend layer. This decoupled design ensures that the core logic execution remains stable while allowing instructors to adapt the simulation to custom laboratory setups.

## Method validation

The simulation method described herein has been deployed in the Industrial Automation Laboratory at the University of Jordan over two consecutive semesters (Fall 2024, Spring 2025), supporting approximately 60 undergraduate mechatronics engineering students. In the course workflow, students first complete virtual simulation tasks at home, then progress to physical laboratory sessions. Typical assigned tasks include: (i) implementing start/stop motor control with indicator lamps, (ii) designing a four-state traffic light sequencer with pedestrian crossing logic, (iii) programming elevator floor selection and direction control, and (iv) automating a filling machine with level sensing and conveyor coordination. Informal instructor observations indicate that students who engaged with the virtual preparation stage required less time troubleshooting hardware connections during physical laboratory sessions and demonstrated increased confidence in ladder logic debugging. A formal assessment of learning outcomes via pre/post surveys and performance metrics is being prepared as a companion education-impact manuscript.

To validate functional equivalence between the virtual and physical laboratories, we executed a set of four representative automation tasks covering all four experimental modules: basic I/O logic, traffic light sequencing, elevator control with floor selection and safety interlocks, and filling machine operation in both automatic and manual modes. These tasks were selected to exercise core PLC programming constructs exactly as implemented in the physical laboratory [10].

### Validation protocol

For each experimental task, we tested two distinct ladder programs: (i) an author-prepared reference solution and (ii) a simplified variant representing typical student code. Both the virtual PLC (Unity) and physical PLC (LS Electric XEC-DR32H) [14] logged I/O state transitions with millisecond-resolution timestamps. Functional equivalence was defined as output state transition times matching within a ±10ms tolerance window, accounting for minor scan-cycle variations.

In all test cases (4 tasks × 2 program variants), the virtual system produced I/O state transitions matching the physical laboratory within the defined tolerance. No logic evaluation errors or unhandled edge cases were observed within the tested program space. Table 3 presents a representative I/O transition log for the Traffic Light task, showing transition times for the North-South green light output (%QX0.0 in physical PLC, labeled P20 in virtual editor) executing a repeating 4s-OFF / 2s-ON cycle. Both systems confirmed alignment within the ±10ms tolerance.

**Table 3:** . Representative I/O transition log for Traffic Light task (North-South Green Light).

Transition #	Event	Virtual Time (ms)	Physical Time (ms)	Deviation (ms)
1	OFF → ON (after 4s delay)	4003	4008	5
2	ON → OFF (after 2s ON)	6011	6017	6
3	OFF → ON (cycle repeat)	10005	10012	7
4	ON → OFF (after 2s ON)	12009	12015	6

### Scan cycle performance

The virtual PLC operates with a slightly different scan cycle than the physical PLC due to differences in software architecture. For the educational tasks validated in this work, which focus on event-driven logic and second-scale timing, this difference does not affect functional behavior or learning outcomes. All validated state transitions matched within the tolerance defined for functional equivalence. The conducted tasks are described as follows.

### Task 1 — I/O simulation

**Fig. 5:**
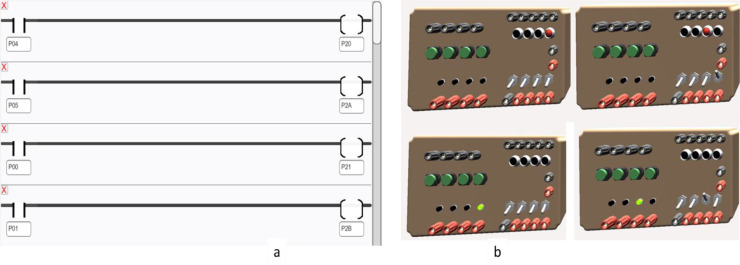
I/O Testing, showing a. I/O task testing code and b. simulation results.

This task verifies the ladder logic code by simulating input signals and observing the corresponding output behavior. In the ladder diagram programming interface, the left column represents inputs, while the right column corresponds to outputs. The Info panel helps identify the specific addresses assigned to each input and output device. To validate the program, the user must first compile the logic using the 'Build' button and then execute it using the 'Run' button. Figure 5a shows a sample code that turns on LEDs P20 and P21 when both a pushbutton (P04) and a toggle switch (P00) are activated. Additionally, socket indication LEDs P2A and P2B are tested similarly. Figure 5b presents the simulation results, confirming the correct operation of the I/O logic.

### Task 2 —Traffic light simulation

The traffic light simulation is designed to test proper timing sequences critical for preventing vehicle collisions. When the ladder logic is programmed correctly with appropriate delays between traffic signal transitions, vehicles proceed safely, moving through green and yellow lights and stopping at red. The Timer (TMR) instruction plays a central role in this experiment. It starts from zero and increments its accumulated value when the input condition is true, eventually reaching the preset value and switching its state to true. If the input becomes false, the timer retains the accumulated value and reverts to false once the reset condition is triggered.

**Fig. 6:**
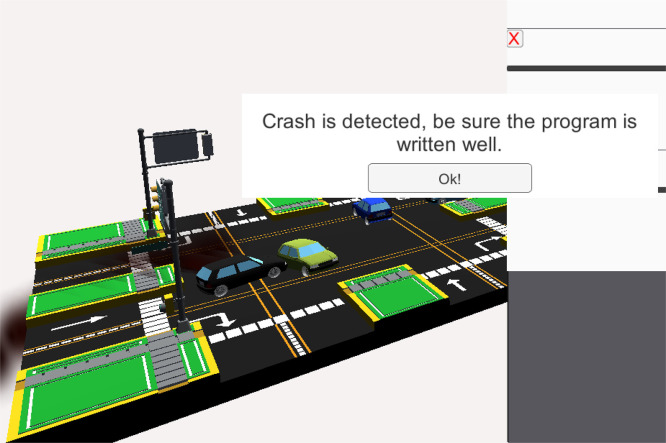
Traffic light simulation task, showing an incorrect code result.

A key educational feature of the task is its built-in error detection mechanism. If students configure the timing incorrectly, such as overlapping green signals for conflicting directions, the simulator identifies the fault, triggers a simulated crash scenario, and displays an error message. This immediate feedback allows students to recognize potentially hazardous situations and understand the real-world consequences of programming errors. An example of such an error message and resulting crash scenario is depicted in Fig. 6.

### Task 3 —Elevator simulation

This task evaluates the correct use of proximity sensors and logic conditions to control elevator movement safely between floors, preventing motor burnout and mechanical collisions. The elevator is expected to respond accurately to floor requests initiated by push buttons and to stop precisely when it reaches the designated floor, as detected by the proximity sensors.

**Fig. 7:**
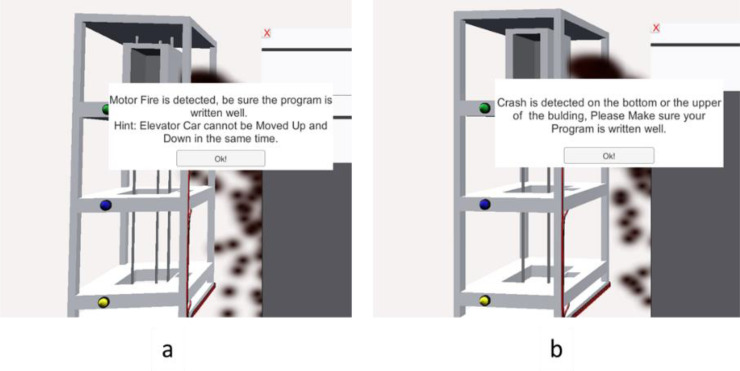
Elevator testing task, showing a. motor simulation error and b. elevator crash error.

To highlight common programming mistakes, several incorrect ladder logic scenarios were tested. In one case, both 'motor up' and 'motor down' outputs were activated simultaneously, which in a real system could lead to motor overheating or failure. The simulator detected this conflict and triggered a 'Motor Fire' error, as shown in Fig. 7a. In another test, logic was written without proper stop conditions, causing the elevator to move continuously without halting at any floor. This led to the elevator crashing into the upper or lower bounds of the shaft, simulating a mechanical failure and displaying a 'Crash Error' message, as illustrated in Fig. 7b. These scenarios provide valuable learning experiences by allowing students to observe the consequences of faulty logic in a safe, virtual environment.

### Task 4 —Filling machine simulation

The filling machine operates in two modes, automatic and manual, determined by the position of the selector switch on the control panel. When set to Mode A, the system runs automatically; in Mode B, it operates under manual control. The process begins when the operator presses the Start button, activating the conveyor belt. The conveyor continues until a proximity sensor detects a container, at which point the belt stops and the filling sequence begins. A second proximity sensor monitors the fill level, and once the container is full, the system responds according to the selected mode: in automatic mode, the conveyor restarts and the cycle repeats; in manual mode, the system pauses until the Start button is pressed again.

**Fig. 8:**
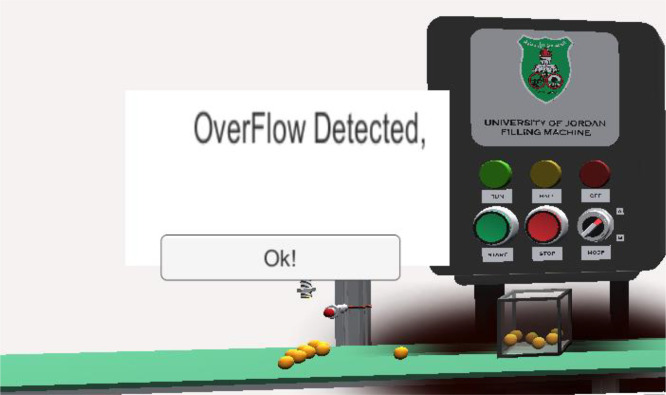
Example of a simulation error in the filling machine kit.

To enhance learning, the simulator incorporates error detection. For instance, if the logic fails to stop the filling process when the container is full, causing an overFlow, an error message is triggered to alert the user. This feature allows students to identify and correct faults related to sensor feedback and process control. A visual example of the overfill error message is shown in Fig. 8.

## Conclusion

This paper presents a modular PLC simulation method that enables exact virtual replication of a cost-effective industrial automation laboratory. The method preserves the input/output architecture, control logic scope, and operational behavior of the physical setup, ensuring that programs developed in simulation produce identical I/O responses when deployed on hardware. Validation across four representative automation tasks confirmed complete functional equivalence between virtual and physical environments. Also the method shows its ability to detect programming faults and simulate common errors, displaying appropriate error messages to aid in debugging and learning. This allows students to prepare for laboratory sessions by writing and testing pre-laboratory code in a virtual environment. This pre-laboratory practice enables more effective use of physical laboratory time, as students arrive with a clearer understanding of the tasks and potential issues. As a result, the method offers a hardware-free, technically faithful alternative for PLC programming development, testing, and training.

## Limitations

The simulation method is specifically designed to replicate the logic behavior and I/O response of a modular educational PLC laboratory comprising four standard experiments: basic I/O, traffic light control, elevator operation, and filling machine automation. It is not intended to model industrial-grade communication protocols (e.g., Modbus, PROFIBUS), analog I/O, or hard real-time performance. Additionally, the ladder logic interpreter supports a core set of relay-based instructions sufficient for the target experiments but does not implement the full instruction set of commercial PLCs. These constraints reflect the method’s focus on functional equivalence within the scope of the original hardware platform, rather than general-purpose industrial simulation.

## Ethics statements

“Not applicable”.

## CRediT author statement

**Musa Al-Yaman and Ahmad Birawi**: Conceptualization, Methodology, Supervision, Resources, Writing- Reviewing and Editing. **Dana Alswaiti** and **Adham Alsharqawi**: Visualization, Resources, Experimentation, Validity tests, Writing- Original draft preparation. **Majid Al-Taee**: Validation, Writing- Reviewing and Editing.

## Declaration of competing interest

The authors declare that they have no known competing financial interests or personal relationships that could have appeared to influence the work reported in this paper.

## Figures and Tables

**Table A1:** Basic I/O experiment.

Virtual Signal	Physical Address	Type	Description
btn_start	%IX0.0	Input	Start button (NO contact)
btn_stop	%IX0.1	Input	Stop button (NC contact)
lamp_run	%QX0.0	Output	Green indicator lamp
lamp_fault	%QX0.1	Output	Red indicator lamp

**Table A2:** Traffic light experiment.

Virtual Signal	Physical Address	Type	Description
sensor_vehicle	%IX1.0	Input	Vehicle detection sensor
light_NS_red	%QX1.0	Output	North-South red lamp
light_NS_green	%QX1.1	Output	North-South green lamp
light_EW_red	%QX1.2	Output	East-West red lamp
light_EW_green	%QX1.3	Output	East-West green lamp

**Table A3:** Elevator experiment.

Virtual Signal	Physical Address	Type	Description
btn_floor_2	%IX2.0	Input	Floor 2 call button
sensor_floor_1	%IX2.1	Input	Floor 1 proximity sensor
sensor_floor_2	%IX2.2	Input	Floor 2 proximity sensor
motor_up	%QX2.0	Output	Elevator motor (up)
motor_down	%QX2.1	Output	Elevator motor (down)

**Table A4:** Filling machine experiment.

Virtual Signal	Physical Address	Type	Description
selector_mode	%IX3.0	Input	Mode selector (A=Auto, B=Manual)
sensor_container	%IX3.1	Input	Container detection sensor
sensor_full	%IX3.2	Input	Tank full level sensor
conveyor_motor	%QX3.0	Output	Conveyor belt motor
valve_fill	%QX3.1	Output	Filling solenoid valve

## Data Availability

Data will be made available on request.

## Appendix: Experiments I/O Mapping Tables

Tables A1, A2, A3, A4
